# Supplementary material for: Maize PPR278 Functions in Mitochondrial RNA Splicing and Editing
Source: Int J Mol Sci. 2022 Mar 11;23(6):3035. doi: 10.3390/ijms23063035 (PMC8949463; doi:10.3390/ijms23063035)
Supplement: Supplementary file 1 [file ijms-23-03035-s001.zip › ijms-1587245-supplementary/Supplementary Figures.pdf]

Supplementary Figures

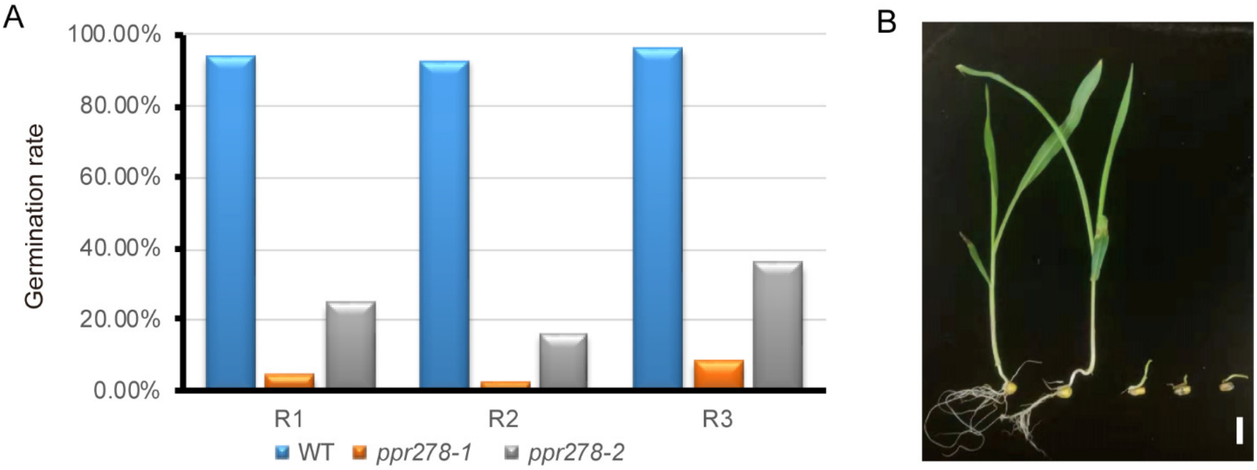

Figure S1. Phenotype of *ppr278* seedlings. (**A**) Analysis of the germination rate of WT and *ppr278-1* and *ppr278-2* mutant seeds. R1, R2, and R3 represent replicates 1, 2, and 3, respectively. (**B**) Seeding development status from heterozygous ears (*PPR278*/*ppr278-1* and *PPR278*/*ppr278-2*) 10 days after sowing (DAS), from left to right: normal kernel of *PPR278-1* and normal kernel of *ppr278-2*, *ppr278-1*, *ppr278-1*, and *ppr278-2* mutants. Bar = 10 mm.

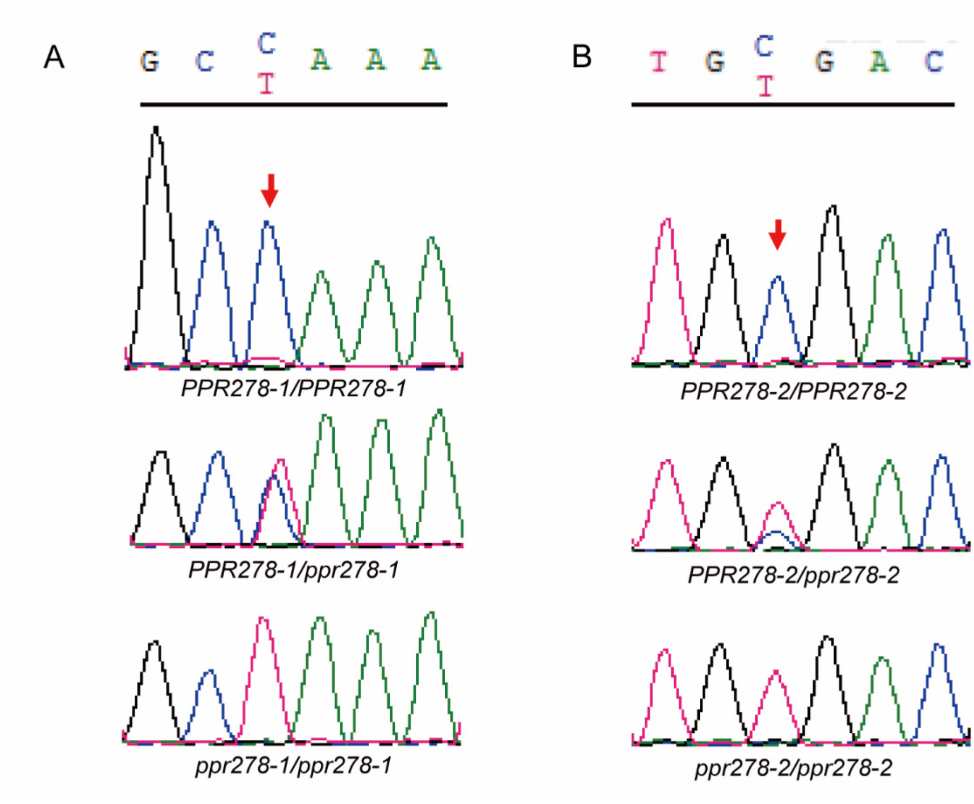

Figure S2. Co-isolation test of *ppr278*. (**A**,**B**). Two pairs of mutant site-spanning primers were designed to test the relationship between the genotype and phenotype in *ppr278-1* and *ppr278-2* mutants.

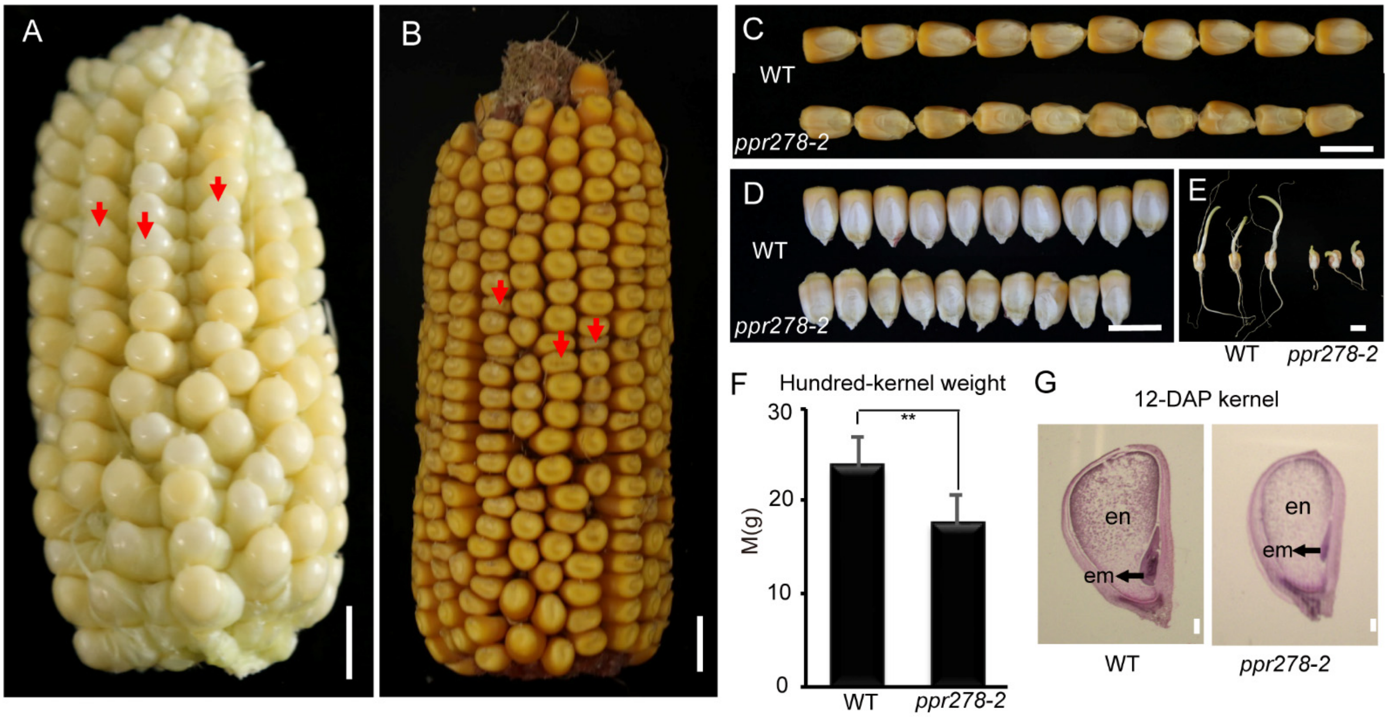

Figure S3. Phenotype of *ppr278-2* mutants was similar to *ppr278-1* mutants. (**A**,**B**) Ears of self-pollinated heterozygous *ppr278-2* plants at 16 days after pollination (16 DAP) (**A**) and at maturity (**B**). Red arrows indicate the mutant kernels. Bar = 1 cm. (**C**,**D**) Comparison of the length (**C**) and width (**D**) of mature seeds between the WT and the *ppr278-2* mutant. Bar = 1 cm. (**E**). Germination of WT and *ppr278-2* mutant seeds after dark culture for 2 days in an incubator. Bar = 1 cm. (**F**). Comparison of the 100-kernel weight of fully mature WT and mutant *ppr278-2* plants. Values and error bars represent the mean and standard deviation of three biological replicates. ***, $P < 0.001$ (*t*-test), representing a significant difference. (**G**). Longitudinal section of WT (left) and *ppr278-2* mutant kernels (right) at 12 DAP. en: endosperm, em: embryo. Bar = 0.5 mm.

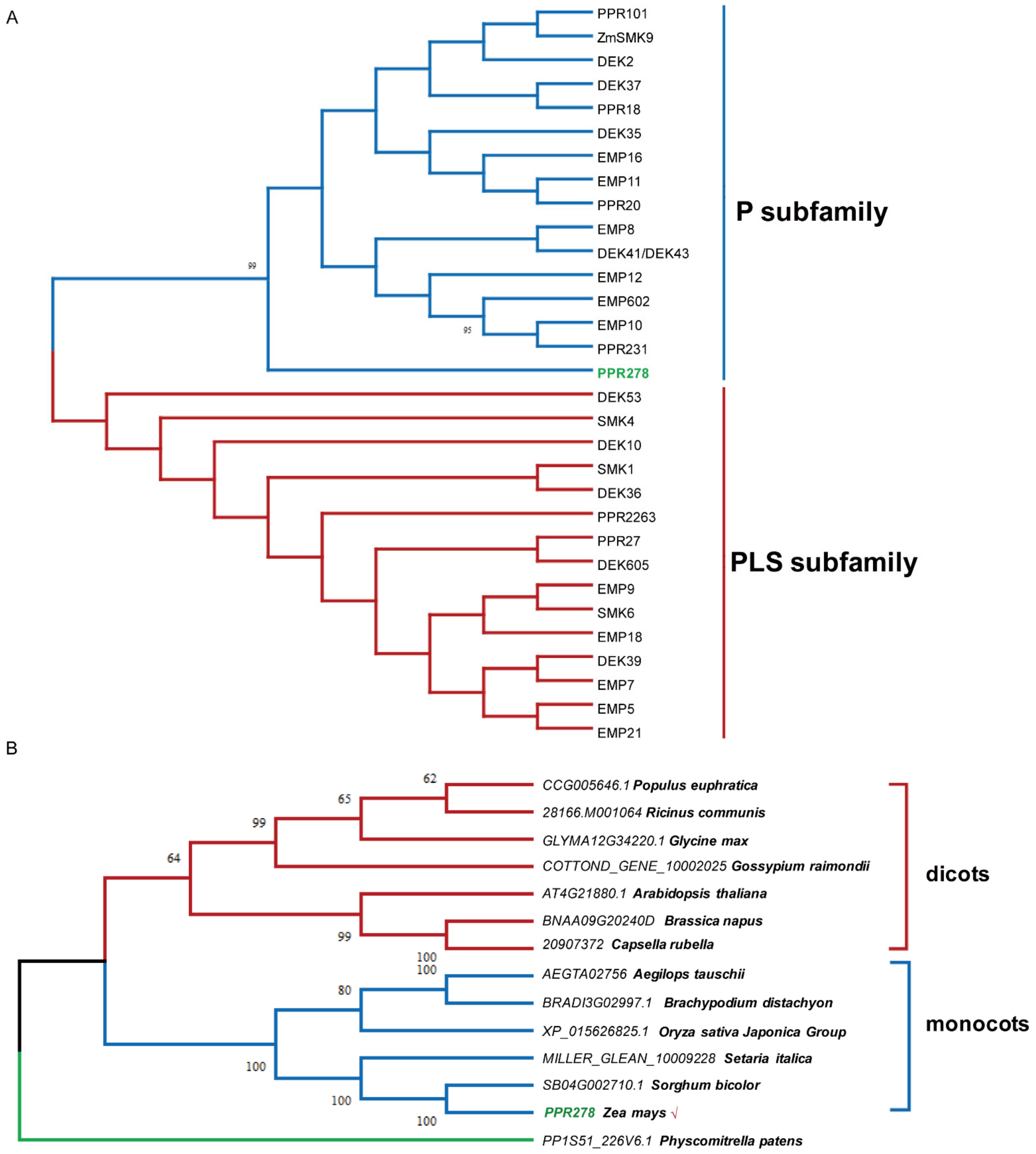

Figure S4. Phylogenetic analysis of PPR278. Phylogenetic analysis of PPR278 protein with published PPR members in maize (**A**) and with homologs in other species (**B**).

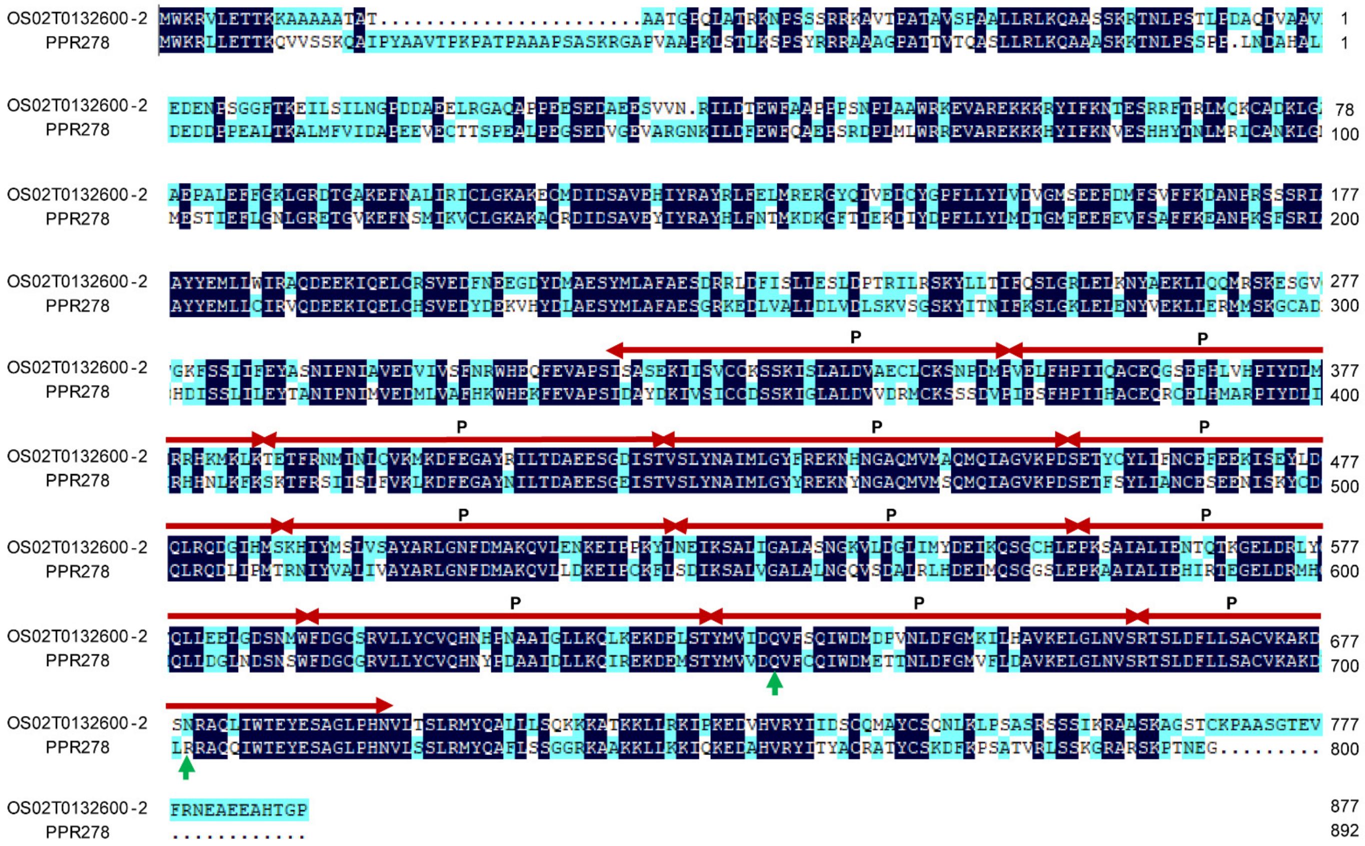

Figure S5. Amino acid sequence alignment of PPR278.Alignment of the amino acid sequence of PPR278 and a putative homolog in rice. Dual red arrows represent the P motifs, and green arrows represent mutant sites.

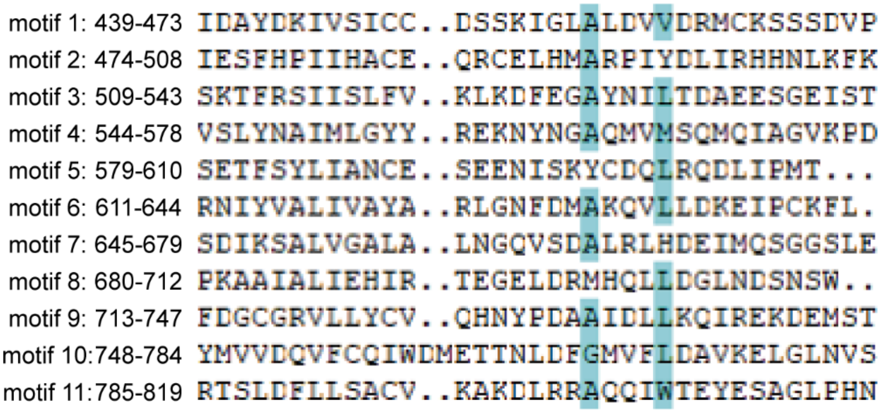

Figure S6. PPR278 contains 11 atypical P motifs. Amino acid sequence alignment of the P motifs in PPR278. The conserved amino acids are shaded greenish-blue.

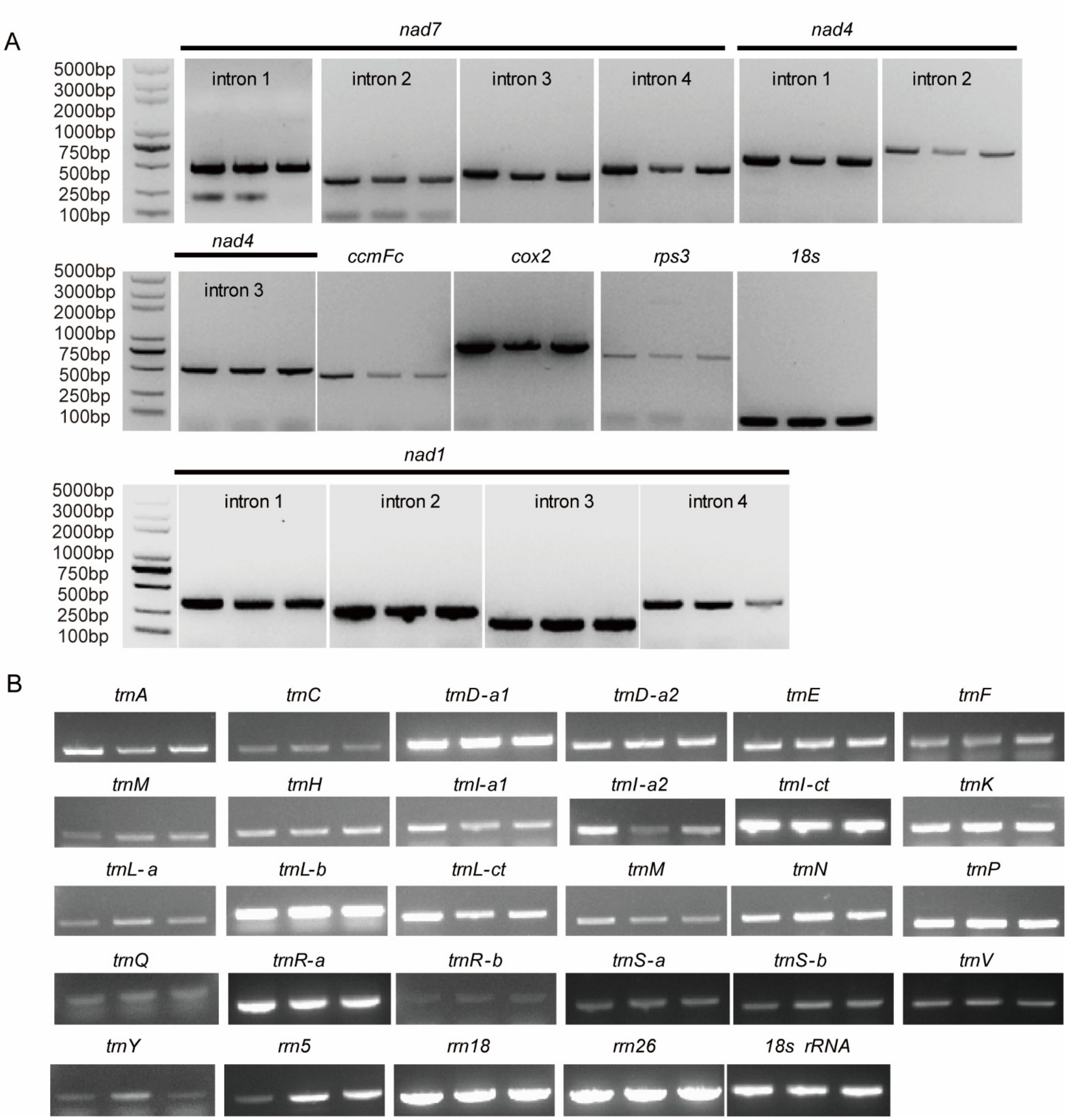

Figure S7. RT-PCR analysis of mitochondrial genes. (**A**) RT-PCR analysis of other group Ⅱ genes except *nad2* and *nad5* intron splicing efficiency in WT, *ppr278-1* and *ppr278-2* respectively. (**B**) Comparison of mitochondrial tRNAs and rRNAs levels between WT and *ppr278* by RT-PCR. Every lane from the left to right represents WT, *ppr278-1*, *ppr278-2* respectively. A DNA marker is shown in the farther left lane.

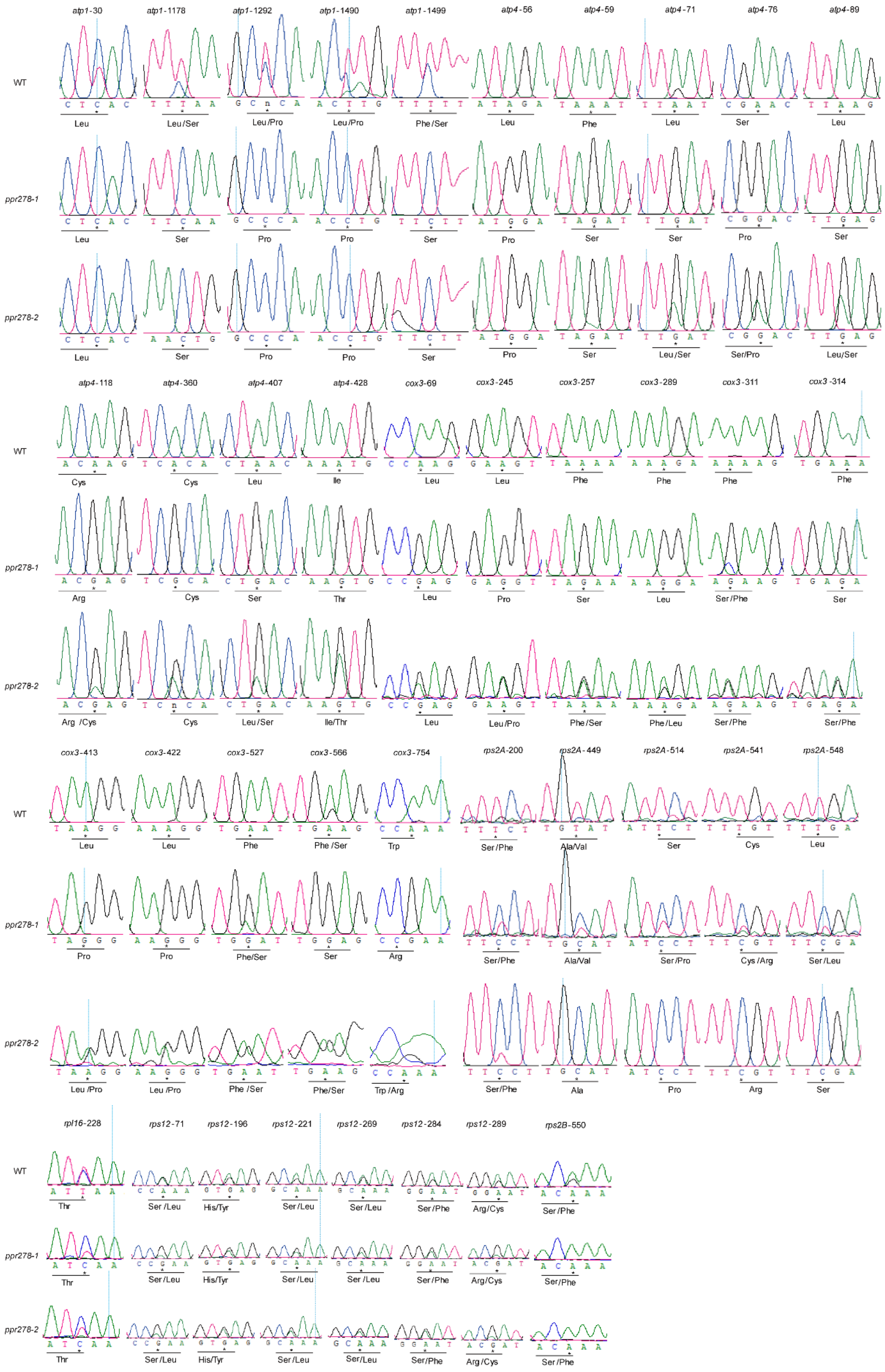

Figure S8. Direct comparison of C-to-U RNA editing sites in mitochondrial genes *atp1*, *atp4*, *cox3*, *rps2A*, *rps2B*, *rps12* and *rpl16* in WT and *ppr278* plants. "*n*" stands for the peak of heterozygous genotype (C/U) in the same editing site. "*" stands for the C-to-U editing site.

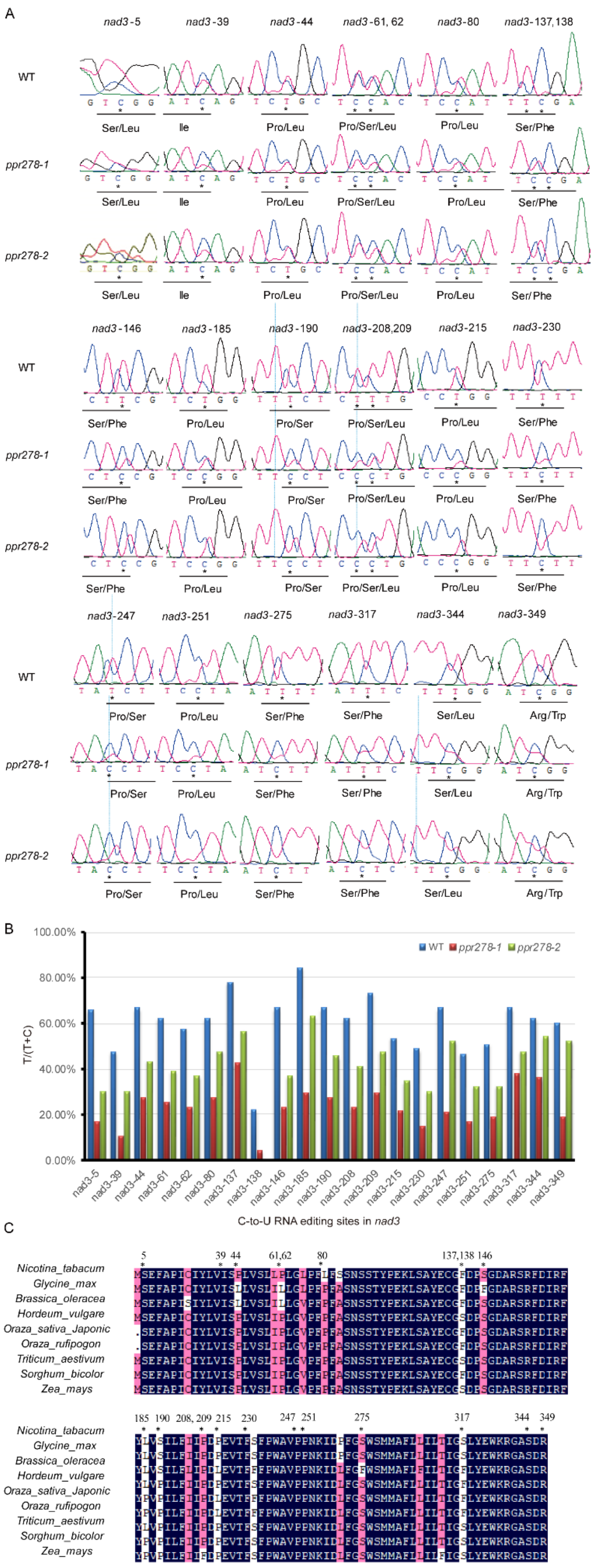

Figure S9. Decreased RNA editing efficiency of *nad3* transcripts in the *ppr278* mutant. (**A**) Direct comparison of C-to-U RNA editing sites in *nad3* in WT and *ppr278* plants. (**B**) Statistics of C-to-U RNA editing efficiency. (**C**) Alignment of *nad3* amino acid sequences in different species. "*" stands for the C-to-U editing site.

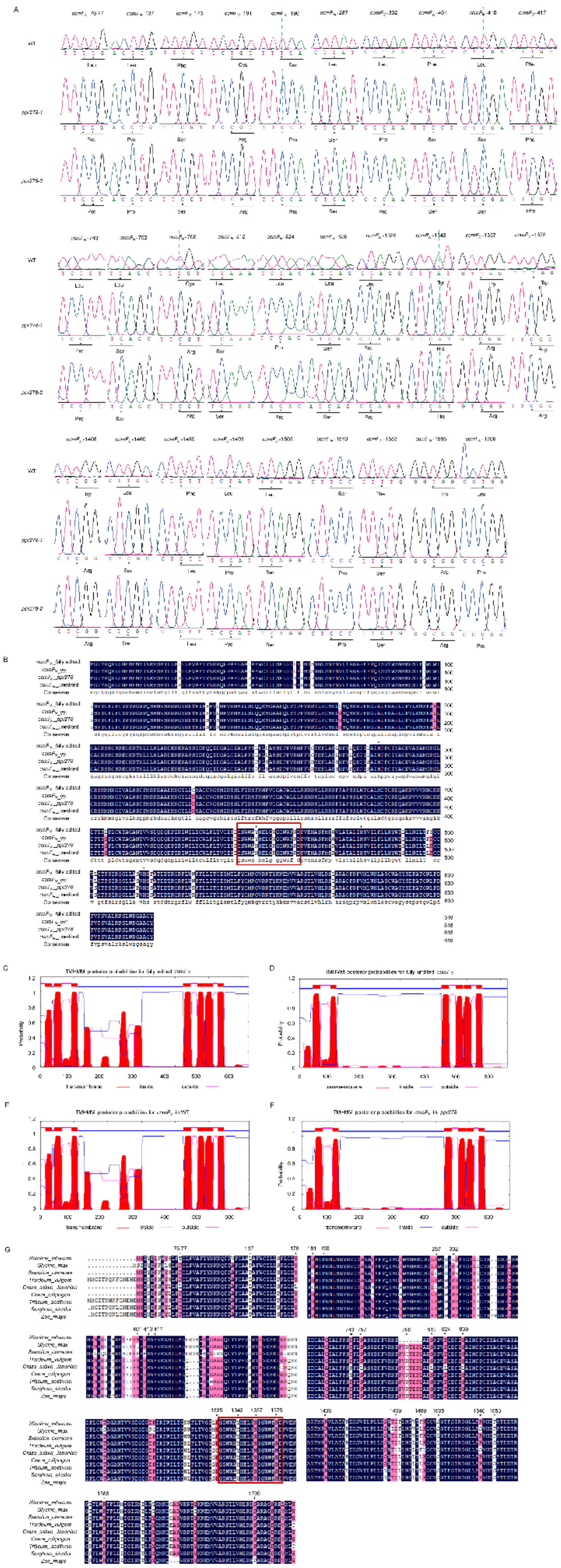

Figure S10. Abolished RNA editing of *ccmFN* transcripts in the *ppr278* mutants. (**A**) Direct comparison of C-to-U RNA editing sites in *ccmFN* in WT and *ppr278* plants. (**B**) Comparison of different C-to-U editing states in *ccmFN*. The white or red background represents amino acids substitution sites in ccmFN, the red rectangle represents the WWD domain, and the stars represent the conserved

histidines (His). (**C**–**F**) The predicted transmembrane domain of different C-to-U RNA editing states of *ccmF*N. (**C**–**D**) All C-to-U RNA editing sites are fully edited (**C**) and unedited (**D**). (**E**–**F**). Two kinds of editing states in WT (**E**) and *ppr278* (**F**) plants. (**G**) Alignment of *ccmF*N amino acid sequences in different species. "*n*" stands for the peak of heterozygous genotype (C/U) in the same editing site. "*" stands for the C-to-U editing site.

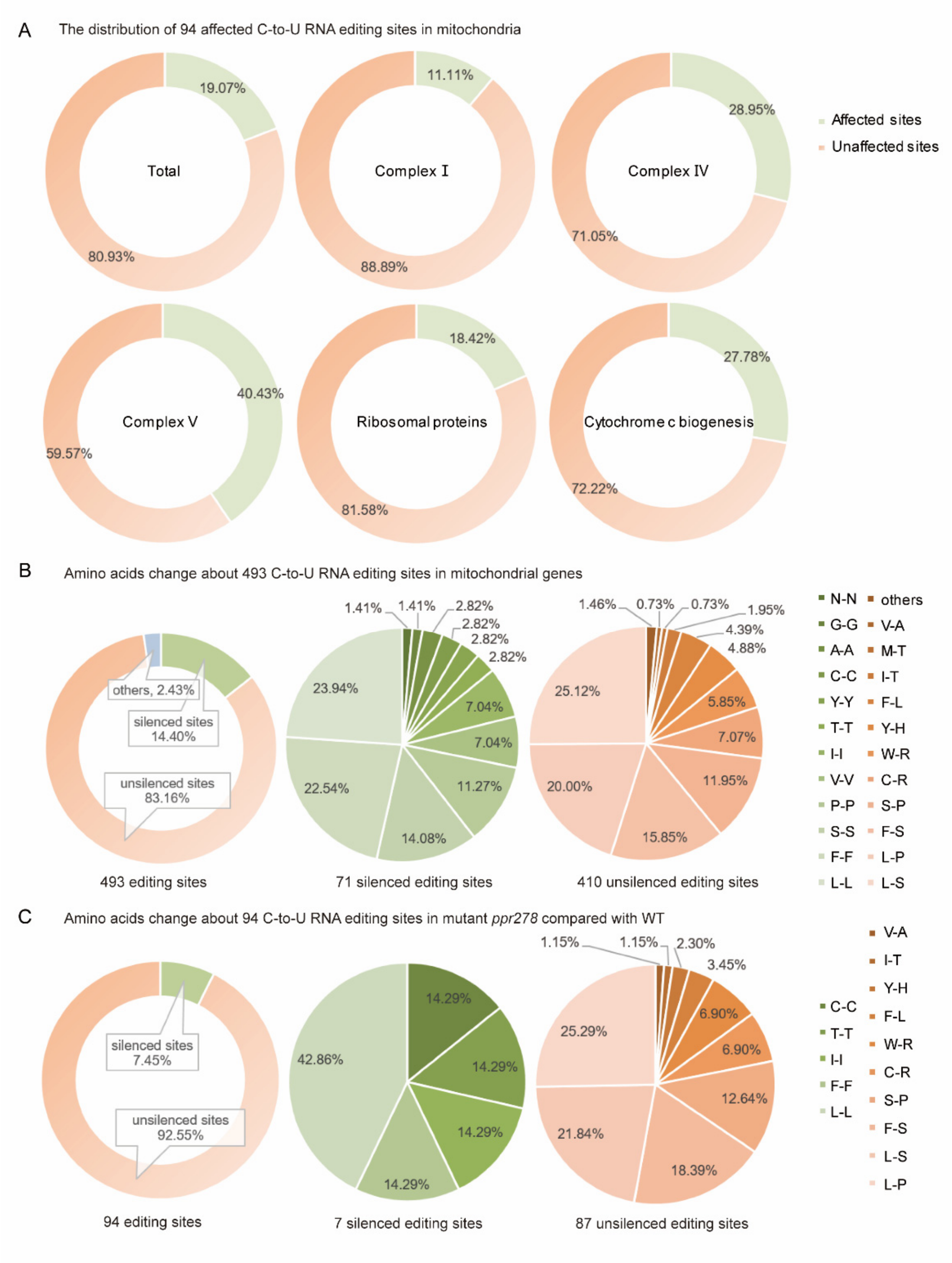

Figure S11. Analysis of amino acid changes related to C-to-U RNA editing of mitochondrial genes. (**A**) The percentage of C-to-U RNA editing in 94 affected sites in the mitochondria genome, complex Ⅰ, Ⅳ, and Ⅴ, cytochrome *c* biogenesis, and ribosomal proteins. (**B**) Amino acid changes at 493 C-to-U RNA editing sites in the mitochondrial genome. "Other" means that C-to-U editing events occurred in untranslated regions. Silenced sites mean that no amino acid change occurred after C-to-U editing. Every pie chart from the left to right represents the statistics of 493 editing sites, 71 silenced sites, and 410 unsilenced sites. (**C**) Amino acid changes at 94 C-to-U RNA editing sites from fully edited to fully unedited states. Every pie chart from the left to right represents the statistics of 94 affected editing sites, 7 silenced sites, and 87 unsilenced sites. 0.
